# A Study of Single Nucleotide Polymorphisms of the *SLC19A1/RFC1* Gene in Subjects with Autism Spectrum Disorder

**DOI:** 10.3390/ijms17050772

**Published:** 2016-05-19

**Authors:** Naila Al Mahmuda, Shigeru Yokoyama, Jian-Jun Huang, Li Liu, Toshio Munesue, Hideo Nakatani, Kenshi Hayashi, Kunimasa Yagi, Masakazu Yamagishi, Haruhiro Higashida

**Affiliations:** 1 Research Center for Child Mental Development, Kanazawa University, Kanazawa 920-8640, Japan; shigeruy@med.kanazawa-u.ac.jp (S.Y.); jianjun453@163.com (J.-J.H.); liuli011258@sina.com (L.L.); munesue@med.kanazawa-u.ac.jp (T.M.); 2 Division of Neuroscience, Kanazawa University Graduate School of Medical Science, Kanazawa 920-8640, Japan; nak@yd5.so-net.ne.jp; 3 Division of Cardiovascular Medicine, Kanazawa University Graduate School of Medical Science, Kanazawa 920-8640, Japan; kenshi@med.kanazawa-u.ac.jp (K.H.); myamagi@med.kanazawa-u.ac.jp (M.Y.); 4 Medical Education Research Center, Kanazawa University Graduate School of Medical Science, Kanazawa 920-8640, Japan; diabe@med.kanazawa-u.ac.jp

**Keywords:** autism spectrum disorder, reduced folate carrier, single nucleotide polymorphism

## Abstract

Autism Spectrum Disorder (ASD) is a group of neurodevelopmental disorders with complex genetic etiology. Recent studies have indicated that children with ASD may have altered folate or methionine metabolism, suggesting that the folate–methionine cycle may play a key role in the etiology of ASD. *SLC19A1*, also referred to as reduced folate carrier 1 (*RFC1*), is a member of the solute carrier group of transporters and is one of the key enzymes in the folate metabolism pathway. Findings from multiple genomic screens suggest the presence of an autism susceptibility locus on chromosome 21q22.3, which includes *SLC19A1*. Therefore, we performed a case-control study in a Japanese population. In this study, DNA samples obtained from 147 ASD patients at the Kanazawa University Hospital in Japan and 150 unrelated healthy Japanese volunteers were examined by the sequence-specific primer-polymerase chain reaction method pooled with fluorescence correlation spectroscopy. *p* < 0.05 was considered to represent a statistically significant outcome. Of 13 single nucleotide polymorphisms (SNPs) examined, a significant *p*-value was obtained for AA genotype of one SNP (*rs1023159*, OR = 0.39, 95% CI = 0.16–0.91, *p* = 0.0394; Fisher’s exact test). Despite some conflicting results, our findings supported a role for the polymorphism *rs1023159* of the *SLC19A1* gene, alone or in combination, as a risk factor for ASD. However, the findings were not consistent after multiple testing corrections. In conclusion, although our results supported a role of the *SLC19A1* gene in the etiology of ASD, it was not a significant risk factor for the ASD samples analyzed in this study.

## 1. Introduction

Autism spectrum disorder (ASD) is a devastating neurodevelopmental disorder with a complex biological basis and is thought to involve multiple and variable gene–environment interactions. ASD is characterized by social impairments, communication problems, and restricted repetitive behaviors [1]. Most candidate genes currently implicated in ASD are involved in neurodevelopmental pathways, social-emotional behavior, or sex or neuropeptide hormonal signaling [2].

The *SLC19A1* gene on human chromosome 21q22.3 [3] encodes one of the key enzymes in the folate metabolism pathway. *SLC19A1*, also referred to as reduced folate carrier 1 (RFC1), functions as a bidirectional anion exchanger, accepting folate cofactors and exporting various organic anions. *SLC19A1* has five exons that contain the total open reading frame (ORF) [4,5,6]. The ORF of human *SLC19A1* cDNA encodes a protein with 12 transmembrane domains and a single *N*-glycosylation site [3,7,8,9]. *SLC19A1* mRNA is detectable in all human tissues [10].

Recent studies indicated that children with ASD may have changed folate or methionine metabolism, suggesting that the folate–methionine cycle may play an important role in the etiology of ASD [11]. Many important genes, including *SLC19A1*, are involved in the folate metabolism pathway and their roles in human diseases, such as gastric and esophageal cancers, have been studied in depth [12,13]. A marginal association with ASD was identified for a 19-bp deletion in the dihydrofolate reductase (*DHFR*) gene (odds ratio (OR): 2.69; 95% CI: 1.00–7.28; *p* < 0.05), which is involved in folate metabolism [14]. Common variants of the decreased folate carrier (*RFC*) and methylene tetrahydrofolate reductase (*MTHFR*) genes conferred increased susceptibility to ASD, suggesting a potential etiological role of impaired folate-dependent one-carbon metabolism in susceptibility to ASD [15].

However, the findings for genes involved in folate transport have been inconsistent between reports. Although the largest study to date found an important association between the *SLC19A1* gene and ASD [15], a subsequent study failed to replicate this finding [16]. Other studies have not identified any mutations in genes included in folate transport in ASD populations [17,18,19].

Here, we hypothesized that genetic variants in *SLC19A1* may play a role in the pathways that are altered in ASD and can therefore be considered candidate genes for testing in ASD patients. We performed a case-control study of 13 genetic variations to assess the involvement of *SLC19A1* in ASD. The study was performed in a Japanese population, in which genetic variants of *CD38* and *BST-1/CD157* were reported to be associated with increased risk of ASD [20,21].

## 2. Results

**Figure 1:**
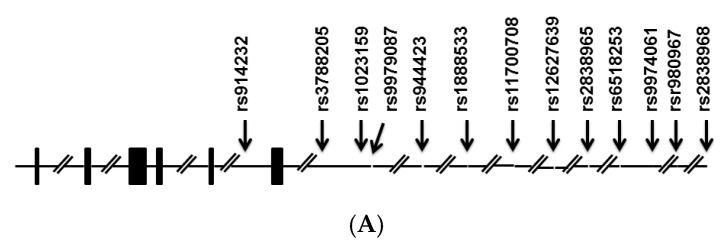

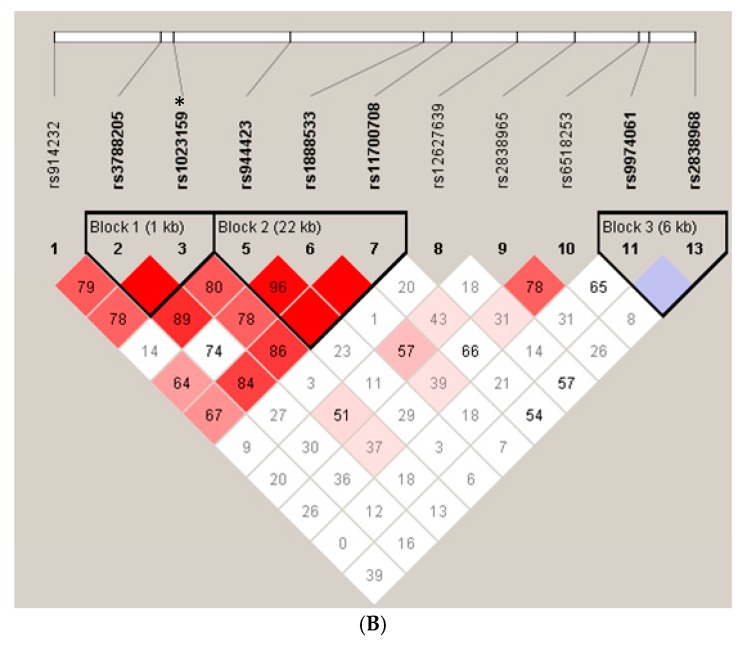
The genomic structure of *SLC19A1* (**A**). Bars, exons. Arrows, positions of single nucleotide polymorphisms (SNPs). Linkage disequilibrium plot of SNPs in the samples studied (**B**). Numbers in squares indicate *D*′ values. Reference Number (rs) with asterisk indicates the SNP with *p* < 0.05. The blocks are defined following the four-gamete rule [22]. Explanation of color scheme: If *D*′ < 1 and LOD (log of the likelihood odds ratio) <2, the cell color is white; if *D*′ = 1 and LOD < 2, the cell color is blue; if *D*′ < 1 and LOD ≥ 2, the cell color is shades of pink/red; if *D*′ = 1 and LOD ≥ 2, the cell color is bright red.

Thirteen SNPs were analyzed in this study, five of which (*rs914232*, *rs3788205*, *rs1023159*, *rs944423*, and *rs9979087*) were located in the *SLC19A1* gene region; these were subjected to statistical analysis. The eight other SNPs (*rs1888533*, *rs11700708*, *rs12627639*, *rs2838965*, *rs6518253*, *rs9974061*, *rs9980967*, and *rs2838968*) were located in the adjacent region. Two SNPs (*rs9980967, rs9979087*) were excluded due to insufficient genotyping data. However, there were no significant associations between any of these SNPs and ASD, with the exception of *rs1023159*. As the results suggested a role (*p* = 0.0394; Table 1) of this polymorphism alone or in combination with others as a risk factor for ASD, this SNP was subjected to further analysis. No association was found after multiple testing corrections. Tests of Hardy–Weinberg equilibrium deviations were performed for each marker in two groups of case and control individuals, and polymorphisms showed evidence of deviation from Hardy–Weinberg equilibrium. The genotyping rate was above 95%. LD analysis of these SNPs identified three haplotype blocks, one of which (Block 1; Figure 1) consisted of two SNPs including one (*rs1023159*) with the lowest *p*-value (*p* = 0.0394; Table 1) among those analyzed.

## 3. Discussion

In this population-based case-control study, we investigated the relationship between polymorphisms in the *SLC19A1* gene and risk of ASD in a Japanese population. We identified no significant associations between SNPs of the *SLC19A1* gene and ASD, with the exception of one SNP, although the results eventually did not support a role of the SLC19A1 gene in the etiology of ASD in our sample.

We have also calculated the genotype and allele frequencies of *rs1023159* in Autism Genome Resources Exchange (AGRE) samples (Table 2). The frequency (15.5%) of the AA genotype in AGRE group of ASD cases, although with different ethnicity, was similar to the frequency (13.7%) observed in the group of Japanese controls.

Recent genetic studies recognized the contribution of the *SLC19A1* gene to neural tube defects (NTD) [23,24,25,26,27,28]. It was suggested that the maternal G allele may be a causative genetic risk factor for having a child with ASD independent of the child’s genotype [29]. In case-control analysis, a significant increase in the *SLC19A1* G allele frequency was discovered among mothers of children with ASD, but not among affected children, and analysis of the *SLC19A1* A80G genotype within family trios discovered that the maternal G allele was allied with a significant increase in risk of ASD, whereas the inherited genotype of the children was not [29].

Evidence indicates that expression of *SLC19A1* in the intestine is subject to adaptive regulation in response to folate status [30]. Folic acid is the inactive, oxidized form of folate compounds that is important for many physiological systems of the body. Folate is the major one-carbon donor for *de novo* nucleotide synthesis for DNA replication and also for remethylation of homocysteine to methionine for essential methylation reactions [29]. The folate cycle interacts with the methionine cycle as well as the tetrahydrobiopterin construction and salvage pathways. Insufficiencies in folate can lead to anomalies in these pathways [31]. The methionine cycle is important for DNA methylation [15], a process that is important in regulating gene expression. Folate deficiency during various significant stages of fetal and infantile development upsets structural and functional alteration of the brain [32].

The reduced folate carrier is the principal mechanism by which folates and antifolates are delivered to mammalian cells and tissues [33]. As folate transport across cell membranes is mediated in part by the RFC, variants within this gene may affect the disease risk via an effect on folate and/or homocysteine levels [34]. Low levels of RFC could result in a number of pathophysiological states associated with folate deficiency, including cardiovascular disease, fetal anomalies, and neurological disorders [33]. Moretti *et al.* reported a 6-year-old girl with developmental delay, psychomotor regression, seizures, mental retardation, and autistic features associated with low cerebrospinal fluid (CSF) levels of 5-methyltetrahydrofolate, the biologically active form of folate in the CSF and blood [35]. Several studies reported considerably low CSF folate concentrations together with normal serum folate concentrations in children with autism [18,19,35,36,37].

*SLC19A1* is situated on the CSF side of the choroid plexus, where it enables transport of concentrated folate into the CSF [11]. Taken together, variation in *SLC19A1* expression may involve both neuronal structures and metabolism in the Central Nervous System (CNS). Defective transport of folate into the CNS is related to cerebral folate deficiency (CFD), a neurological disorder that is important in diagnosis of children with unexplained neurodevelopmental symptoms, which suggests the possible involvement of the folate-methionine pathway in ASD [31]. Further, early-onset low-functioning autism with neurological deficits has been suggested as a characteristic of children with both autism and CFD [18,19,35,38].

The major limitation of this study was the small sample size, resulting in deviation from Hardy–Weinberg equilibrium and limited power (68%) to reliably detect the role of *SLC19A1* in ASD. We did not recognize any population stratification, admixture, and cryptic relation among the subjects in the present study, which may have contributed to the lack of association in this small sample. Another limitation was the lack of a replication cohort. Further studies with larger sample sizes and/or family-based association testing are needed to clarify the precise role of this gene in ASD. However, our findings were consistent with reports that *SLC19A1* may not contribute to genetic susceptibility to ASD in some populations.

## 4. Materials and Methods

### 4.1. Study Population

The study population consisted of 147 ASD subjects (113 males, 34 females; 15.6 ± 0.6 years old, mean ± s.e.m.) from the Outpatient Psychiatry Department of Kanazawa University Hospital, as described previously [20,39]. All subjects satisfied the Diagnostic and Statistical Mannual of Mental Disorders-IV (DSM-IV) criteria for pervasive developmental ailment and Childhood Autism Rating Scale. Two experienced child psychiatrists established the diagnosis of ASD in all patients based on semi-structured behavioral observations and conversations with the subjects and their parents. The interview structure and clinical records were described previously [20]. One of the following methods was used as an aid to evaluate the autism-specific behaviors and symptoms during interviews with parents: the Asperger Syndrome Diagnostic Interview [40], Autism Diagnostic Interview-Revised (ADI-R) [41], Pervasive Developmental Disorders Autism Society Japan Rating Scale [42], Diagnostic Interview for Social and Communication Disorders [43], or Tokyo Autistic Behavior Scale [44]. A total of 150 individuals were recruited as controls (115 males, 35 females; 23.8 ± 0.3 years old). All patients and controls were Japanese with no non-Japanese parents or grandparents. These controls were part of a stock used frequently for single nucleotide polymorphism (SNP) analysis of ion channels related to arrhythmia at Kanazawa University Heart Center. This study was approved by the ethics committee of Kanazawa University School of Medicine (July 2015), and all participants and/or their caregivers provided informed consent. The study protocol was performed in accordance with the Declaration of Helsinki.

### 4.2. Genotyping

Genomic DNA was extracted as described previously [39] from venous blood samples using a commercial kit (Wizard Genomic DNA Purification kit; Promega, Madison, WI, USA) or from nails using an ISOHAIR DNA extraction kit (Nippon Gene, Tokyo, Japan). The genomic DNA samples were subjected to whole-genome amplification, and SNPs were determined by the sequence-specific primer-polymerase chain reaction (SSP-PCR) method followed by fluorescence correlation spectroscopy as described by Bannai *et al.* [45]. We selected a set of tagging SNPs that capture common variations and linkage disequilibrium (LD) structures across the *SLC19A1* gene using the Tagger program incorporated with Haploview v4.2 software(Broad Institute of MIT and Harvard, Cambridge, MA, USA). The data source for tagging SNPs was the dbSNP database [46] and the HapMap genome browser, release 27 (operated by the National Institutes of Health (NIH), Bethesda, MD, USA) in the JPT (Japanese individuals from Tokyo, Japan), CHB (Han Chinese individuals from Beijing, China), ASW (African ancestry in Southwest USA), and CEU (Utah residents of northern and western European ancestry) populations. Selection of tagging SNPs was based on pairwise tagging only and the minor allele frequency was ≥5% in any one of the different ethnicities.

### 4.3. Statistical Analysis

Genotype and allele frequencies were examined using a contingency table and Fisher’s exact test (GraphPad Prism 6; GraphPad Software, San Diego, CA, USA), and *p* < 0.05 was taken to indicate statistical significance. We also used the method of Nyholt [47], which estimates an “effective number” of independent tests and then adjusts the smallest observed *p*-value using simulation based on this number of tests. In our samples, the estimated effective number for independent tests was 9 and the *p-*value was 0.005. The observed genotype frequency distributions were compared with those expected from the Hardy–Weinberg equilibrium and analyzed by the chi “χ” squared test.

Statistical power was calculated using the Genetic Power Calculator [48,49] assuming a population prevalence of 0.015 for ASD [50], and a *D*’ value of 1 between the marker and disease with a false positive rate of 5%.

## 5. Conclusions

This study showed no evidence supporting a role of the *SLC19A1* gene in the etiology of ASD. The ethnic and cultural background may have influenced the results of our study. However, these findings warrant additional discussion and confirmation in subsequent studies. Further cellular and molecular studies are required to elucidate the precise role of this gene in ASD.

## Figures and Tables

**Table 1:** Genotype and allele frequencies of *rs1023159* at Kanazawa University Hospital for autism spectrum disorder (ASD).

*rs1023159*	Cases	Control	Odds Ratio (95% CI)	*p*
Genotype	(*n* = 144)	(*n* = 146)		
G/G	72 (50.0%)	62 (42.5%)	Reference	
A/G	63 (43.8%)	64 (43.8%)	0.85 (0.52–1.4)	0.5368
A/A	9 (6.3%)	20 (13.7%)	0.39 (**0.16**–**0.91**)	0.0394
Allele	(*n* = 288)	(*n* = 292)		
G	207 (71.9%)	188 (64.4%)	Reference	
A	81 (28.1%)	104 (35.6%)	0.71 (0.50–1.0)	0.0613

CI, confidence interval; *p*-values obtained by Fisher’s exact test are given; *p* < 0.05 is indicated in bold.

**Table 2:** Genotype and allele frequencies of *rs1023159* in KU samples and AGRE samples for autism spectrum disorder (ASD).

*rs1023159*	KU	AGRE
Genotype	(*n* = 144)	(*n* = 191)
G/G	72 (50.0%)	63 (31.4%)
A/G	63 (43.8%)	104 (53.1%)
A/A	9 (6.3%)	30 (15.5%)
Allele	(*n* = 288)	(*n* = 394)
G	207 (71.9%)	230 (58.4%)
A	81 (28.1%)	164 (41.6%)

KU, Kanazawa University; AGRE, Autism Genome Resources Exchange.
